# Chagas Disease Has Not Been Controlled in Ecuador

**DOI:** 10.1371/journal.pone.0158145

**Published:** 2016-06-28

**Authors:** Eric Dumonteil, Claudia Herrera, Luiggi Martini, Mario J. Grijalva, Angel G. Guevara, Jaime A. Costales, H. Marcelo Aguilar, S. Frédérique Brenière, Etienne Waleckx

**Affiliations:** 1 Laboratorio de Parasitología, Centro de Investigaciones Regionales ‘‘Hideyo Noguchi”, Universidad Autónoma de Yucatán, Mérida, Yucatán, México; 2 Department of Tropical Medicine, Vector-Borne Infectious Disease Research Center, School of Public Health and Tropical Medicine, Tulane University, New Orleans, LA, United States of America; 3 Laboratorio de Parasitología “L. Martini y colaboradores”, Guayaquil, Ecuador; 4 Tropical Disease Institute, Department of Biomedical Sciences, Heritage College of Osteopathic Medicine, Ohio University, Athens, OH, United States of America; 5 Center for Infectious and Chronic Disease Research, School of Biological Sciences, Pontifical Catholic University of Ecuador, Quito, Ecuador; 6 Centro de Biomedicina, Carrera de Medicina, Universidad Central del Ecuador, Quito, Ecuador; 7 Unidad de Salud Pública, Instituto Superior de Investigación y Posgrado, Facultad de Ciencias Médicas, Universidad Central del Ecuador, Quito, Ecuador; 8 IRD, UMR INTERTRYP (IRD-CIRAD), Interactions hosts-vectors-parasites environment in the tropical neglected disease due to trypanosomatids, TA A-17/G, Campus international de Baillarguet, Montpellier, France; Albert Einstein College of Medicine, UNITED STATES

A recent study by Cartelle Gestal et al. reported an analysis of data from the Ministry of Public Health on the epidemiological situation of neglected tropical diseases in Ecuador [1]. Based on a misleading definition of Chagas disease cases not corresponding to that of the Ministry of Public Health [2], the authors concluded that the government had mounted successful control campaigns, and as a result Chagas disease (among others) had been effectively controlled as no cases in children under age five had been reported since 2009. Ecuador is thus identified as one of the first countries to control Chagas disease.

While we certainly agree that efforts have been made in terms of Chagas disease surveillance and control campaigns in Ecuador, a more comprehensive analysis of available data, from both the Ministry of Public Health and the literature, provides a very different picture, and the claim that Chagas disease is controlled made by Cartelle Gestal et al. seems largely inadequate and sends an equivocal message which can undermine current control efforts. As mentioned in this study, the Chagas disease control program in the country was formally established in 2003–2004, in response to recommendations from a technical consultation through PAHO/WHO [3] and field studies [4,5]. This consultation and data provided a baseline to prioritize activities. It reported a national seroprevalence of *Trypanosoma cruzi* infection of 1.38%, corresponding to 165–170,000 seropositive patients in the country. Three regions were prioritized: the coastal region (seroprevalence of 1.99%), the Amazon region (1.75%) and the southern highlands (0.65%). The incidence was estimated at 36 cases/100,000 inhabitants/year, resulting in 4,400 new cases each year [3].

Today, the most recent estimates from the WHO suggest the presence of nearly 200,000 seropositive patients and a current incidence of 14 cases/100,000 inhabitants/year [6]. An in depth analysis of the complete records from the Ministry of Public Health from 2004–2014, indicates a total of 915 reported human cases in the country, with a major increase over the years followed by a decrease in the past two years [7]. This increase reflects the efforts at improving the epidemiologic surveillance program, but it is clear that there is still significant underreporting of cases in the country. Indeed, several independent and recent seroprevalence studies in different regions and communities point out relatively high levels of seroprevalence of *T*. *cruzi* infection (ranging from 0.6 to 13.3%), and persistent active parasite transmission, as evidenced by the detection of seropositive children [8–12]. Additionally, there are reports of Chagas disease cases in regions where the Ministry of Public Health has no records of patients, further highlighting current underreporting [5,11,12]. Furthermore, while during the last decade Ecuador has achieved near 100% blood screening coverage for *T*. *cruzi* infection, the 15 participating blood banks regularly report seropositive blood donors to the External Performance Evaluation of Serological Screening Program administered by the Pontifical Catholic University of Ecuador.

The vector control program was effectively started in 2004. However, due to limited human and financial resources, there have been important variations in the geographic coverage of the surveillance and control activities from year to year [7]. Importantly, a total of 12 provinces have not been included in these activities, representing an area larger than the covered provinces. Therefore, the available data do not correspond to a systematic national coverage, and thus still present an incomplete picture of the current transmission of Chagas disease in Ecuador. In the 11 provinces in which surveillance and control activities have been performed, house infestation by triatomines is still observed in many regions [7,13]. While vector control activities have had a significant effect and allowed reducing the infestation level, particularly in coastal Ecuador, these need to be sustained to avoid reinfestation and provide long-term effects. Also, while insecticide spraying may be effective against *Triatoma dimidiata*, a possibly domiciliated species which is poised for elimination in Ecuador, alternative control strategies may be needed against intrusive triatomine species such as *Rhodnius ecuadoriensis* or *Panstrongylus howardi* or for occasional exposure outside of homes [14–19]. Moreover, no formal vector control intervention has been implemented in the Amazon region, where nearly half of the cases of the country seem to originate [7], and active transmission still occurs through triatomine species including *Rhodnius robustus* and *R*. *pictipes* [8,9]. Especially in the Amazon, human activities (deforestation, urbanization) disturb the natural balance between the vectors, their wild hosts and the parasite, favoring the emergence of new transmission cycles in which humans may be included [8,9,11,20].

An accurate description of the situation of Chagas disease in Ecuador should mention that access to diagnosis throughout the country is limited and case detection during the last two decades has been sporadic and geographically restricted. Indeed, only one laboratory in the whole country, at the *Instituto Nacional de Investigación en Salud Pública* (INSPI), performs official confirmation of anti-*T*. *cruzi* seropositivity and releases Nifurtimox for the treament of patients. In fact, we believe that lack of awareness by health care personnel in areas with active vectorial transmission, combined with lack of diagnostic capacity elsewhere in the country, have resulted in a gross under reporting of cases in Ecuador.

Taken together, these data and studies highlight that Chagas disease is all but controlled in Ecuador, contrary to what is stated by Cartelle Gestal et al. While it is clear that disease surveillance and vector control activities from the Ministry of Public Health have improved over the years, these need to (i) reach national coverage to ensure the inclusion of all endemic provinces, and (ii) be sustained to ensure that what has been achieved can result in long-term control of the disease. These represent a clear challenge at a time when the Ministry of Public Health is undergoing major structural reorganization and many of its activities are being decentralized or interrupted. Indeed, there is a decrease in reported human cases and in vector controls activities observed in the past two years in Ecuador [7], which may reflect the interruption of the National Chagas Program and the *Servicio Nacional de Control y Vigilancia de Enfermedades Transmitidas por Vectores Artrópodos* (SNEM) in late 2015. Their actions have not been replaced yet, so that there is currently no Chagas vector control program in the country. This can strongly jeopardize the results achieved so far and may be a lost opportunity to eliminate vectorial transmission with domiciliated vectors in some regions of Ecuador.

Finally, as in many other countries in Latin America, current activities for Chagas disease control in Ecuador still need to improve treatment access and care for Chagas disease patients [21–23] as well as to better understand the importance of congenital transmission in the epidemiology of the disease [9,24]. Thus, control of Chagas disease in the country will only be reached if the programs from the Ministry of Public Health are strengthened and expanded. The National Chagas disease control programs in other Latin America countries such as Brazil, Argentina, or Colombia (among others) can provide key examples of successful strategies for Chagas disease surveillance and control, as well as of the challenges encountered for their implementation. Additionally, research needs to be performed to further expand our understanding of triatomine infestation and *T*. *cruzi* transmission cycles in the different specific endemic areas, to help further tailor surveillance and interventions. More than claiming that Chagas disease is controlled, we need to promote further political commitment to sustain current achievements in Chagas disease surveillance and control in Ecuador and to ensure that the goals of the London declaration on neglected tropical diseases [25] are met in the near future.
